# Mandibular morphology and the Mesolithic–Neolithic transition in Westernmost Iberia

**DOI:** 10.1038/s41598-023-42846-z

**Published:** 2023-10-03

**Authors:** Ricardo Miguel Godinho, Cláudia Umbelino, António Carlos Valera, António Faustino Carvalho, Nuno Bicho, João Cascalheira, Célia Gonçalves, Patricia Smith

**Affiliations:** 1 Interdisciplinary Center for Archaeology and Evolution of Human Behaviour (ICArEHB), Faculdade das Ciências Humanas e Sociais, University of Algarve, Campus Gambelas, 8005-139 Faro, Portugal; 2 Department of Life Sciences, Research Centre for Anthropology and Health, University of Coimbra, Coimbra, Portugal; 3 Era Arqueologia, S.A., Calçada de Santa Catarina, 9C, 1495-705 Cruz Quebrada, Portugal; 4 Centro de Estudos de Arqueologia, Artes e Ciências do Património (CEAACP), F.C.H.S., University of Algarve, Campus de Gambelas, 8000-117 Faro, Portugal; 5 Faculties of Medicine and Dental Medicine and National Natural History Collections, The Hebrew University, Jerusalem, Israel

**Keywords:** Anthropology, Archaeology

## Abstract

Neolithic farming and animal husbandry were first developed in the Near East ~ 10,000 BCE and expanded westwards, reaching westernmost Iberia no later than 5500 BCE. It resulted in major social, cultural, economic and dietary changes. Yet, the impact of this change on human mandibular morphology in Iberia is yet to be assessed, which is regrettable because mandible form is impacted by population history and diet. In this study we used Mesolithic to Chalcolithic Iberian samples to examine the impact of this transition on mandibular morphology. We also compared these samples with a Southern Levantine Chalcolithic population to assess their relationship. Lastly, we assessed dental wear to determine if the morphological differences identified were related to the material properties of the diet. We found differences between samples in mandibular shape but not size, which we attribute to contrasting population histories between Mesolithic and later populations. Some differences in the severity of dental wear were also found between Mesolithic and later Iberian samples, and smaller between the Mesolithic Iberians and southern Levantines. Little relationship was found between wear magnitude and mandibular shape. Altogether, our results show that the Mesolithic–Neolithic Iberian transition resulted in a meaningful change in mandibular morphology, which was likely driven more by population history than by dietary change.

## Introduction

Neolithic farming and animal husbandry were first developed in the Near East about 9000–10,000 BCE^1^ and subsequently expanded throughout Europe via two main routes: via the Danube into central Europe, and via the Mediterranean into Southern and Western Europe^2–7^. Ancient DNA (aDNA) studies have shown that the expansion of this new mode of subsistence was associated with demic diffusion of Near Eastern populations and their flocks that reached Western Iberia (i.e., modern-day Portugal and Galicia) no later than ~ 5500 BCE^2–6,8–11^. This is evident since aDNA studies show marked genetic discontinuity between Iberian Mesolithic foragers and the Neolithic agro-pastoralist populations, despite some level of admixture^12–16^.

The transition from Mesolithic foraging to Neolithic agro-pastoralism also involved profound dietary changes, including the shift from a mechanically more demanding diet in the Mesolithic to a softer diet in the Neolithic and later periods^17,18^. This change in the material properties of foodstuffs has been supported by studies of skeletal morphology from several geographic regions documenting that Mesolithic skulls were generally larger, more robust and differently shaped than Neolithic specimens^19–23^. Studies of dental wear magnitude in western Iberia have also shown that this was more severe in Mesolithic than in Neolithic and Chalcolithic populations, providing direct evidence of decreased masticatory demands from the Neolithic onwards^24,25^. Elsewhere, differences in dental microwear have also been detected between foragers, farmers and pastoralists^26–28^. Stable isotope-based research in this region has reported a transition from diverse Mesolithic diets (reflecting the exploitation of several ecological contexts) with a meaningful component of marine foodstuffs, to less diverse Neolithic diets largely dominated by terrestrial products in which marine products, despite consumed, were less relevant^24,29–33^. Lastly, recent metagenomic analyses have also shown changes in the oral microbiome following the adoption of Neolithic agro-pastoralism in the Central-South Italian Peninsula^34^. Such dietary changes have been shown to result in changes in dental disease patterns in different regions^35–40^.

Previous continental scale studies have shown that diachronic change in cranial morphology across Europe and the Levant is generally consistent with the archaeological record and aDNA results indicative of population movement as well as cultural diffusion^41,42^. Yet such studies are limited in the context of Iberia because they do not include post-Mesolithic specimens, thus precluding analysis of morphological change during the Mesolithic-Neolithic transition in the Peninsula. The only study on Mesolithic and Neolithic crania specific to Iberia is that of Jackes, Lubell and Meiklejohn^43^, who concluded that their findings did not support hypotheses of population discontinuity during this transition. However, these studies focused on cranial measurements and did not include mandibular form. Mandibular morphology has been shown to relate to both population history^19,44,45^ and diet^19–23^, and thus both population and dietary changes should impact Iberian mandibular morphology. Yet, to date, there is only one study on this topic^46^. In that study, the authors found differences in mandibular shape between Iberian Mesolithic foragers and Chalcolithic agro-pastoralists, but could not assess the timing of the changes identified, nor the extent to which they reflected demic change as opposed to dietary change.

Here we address these issues using Geometric Morphometrics (GM) to examine the impact of the Mesolithic–Neolithic transition on Iberian mandibular morphology by comparing local Mesolithic foragers, Neolithic and Chalcolithic agro-pastoralists, and southern Levantine Neolithic and Chalcolithic agro-pastoralists. We also scored dental wear, to assess the contribution of function to the observed morphological differences. Based on aDNA studies and the relationship between mandibular morphology and population history (see above), we expect significant differences between Mesolithic Iberians and Chalcolithic/Neolithic southern Levantines, and test this against a null hypothesis of no differences between these populations. We further hypothesise that post-Mesolithic Iberians are intermediate between the two former populations. Last, we examine the relationship between dental wear and mandibular shape and (considering the relationship between mandibular morphology and diet) expect to find a significant relationship between the two.

## Results

**Figure 1:**
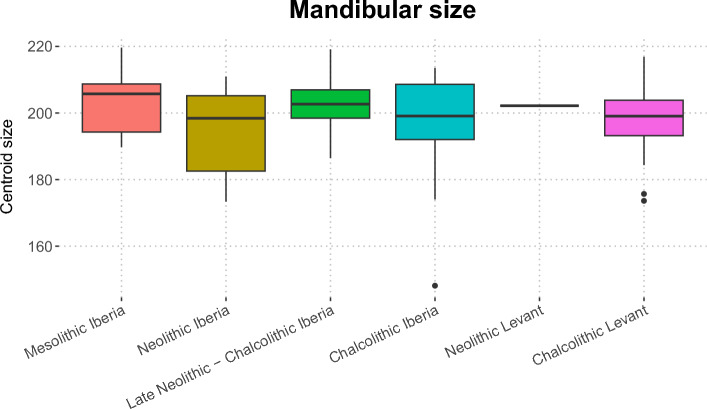
Mandibular size (as assessed using centroid size) of specimens (grouped by chronology and geographic origin). No statistically significant differences were found (see details in the text).

We found no significant differences in mandibular size between groups (Fig. 1; H(5) = 6.5186, *p* = 0.259) but did find significant differences between them in shape. Using PERMANOVA we found that the scores from the first 29 PCs (explaining ~ 95% of the total variance) showed statistically significant shape differences (*p* < 0.0001) between the different groups. Pairwise comparisons revealed highly significant differences between Mesolithic Iberia and all other groups (*p* < 0.01) except for the Late Neolithic—Chalcolithic Iberian sample, where the results were borderline (*p* = 0.051), while the Levantine Chalcolithic group was significantly different from all the Iberian groups (Table 1).

**Table 1:** *P* values of PERMANOVA pairwise post-hoc testing (in both triangles of the matrix).

	Mesolithic Iberia	Neolithic Iberia	Late Neolithic—Chalcolithic Iberia	Chalcolithic Iberia	Chalcolithic Levant
Mesolithic Iberia		**0.007**	0.052	**0.002**	**0.001**
Neolithic Iberia	**0.007**		1.000	0.653	**0.007**
Late Neolithic—Chalcolithic Iberia	0.052	1.000		1.000	**0.016**
Chalcolithic Iberia	**0.002**	0.653	1.000		**0.006**
Chalcolithic Levant	**0.001**	**0.007**	**0.016**	**0.006**	

*P* values are Bonferroni adjusted and highlighted in bold when statistically significant.

**Figure 2:**
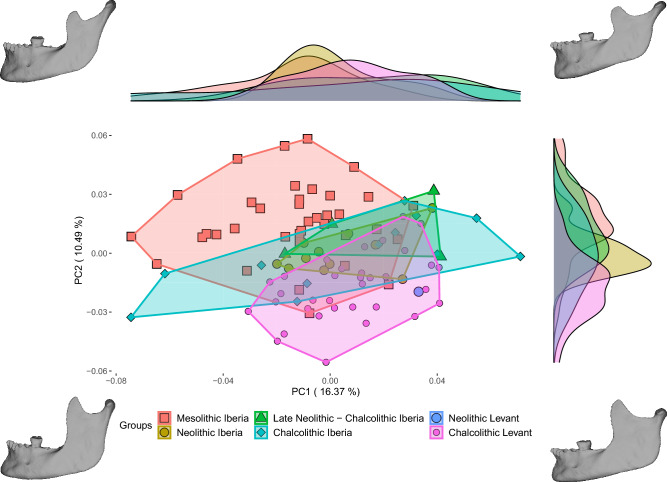
PCA of mandibular shape. Individuals are grouped according to chronology and geographic origin. The Mesolithic Iberian and Chalcolithic Levant groups are morphologically distinct, despite some overlap. The post-Mesolithic Iberian specimens are intermediate between the two extreme previous groups (i.e., Mesolithic Iberia and Chalcolithic Levant), overlapping with both. The mandible insets represent the morphology of the specimens at the extremes of the PC axes (e.g., the mandible at the bottom left corner represents the morphology of a hypothetical specimen located at the negative end of PCs 1 and 2).

Visual assessment of the distribution of individuals along PC1 (16.37% of the variance) and PC2 (10.49% of the variance) shows some overlap between the Mesolithic Iberian and the Chalcolithic Levantine specimens (Fig. 2, SI Fig. 1). Nevertheless, these two groups are significantly different (SI Fig. 2). Iberian post-Mesolithic specimens fall between the Iberian Mesolithic and Levantine Chalcolithic groups, overlapping with both and showing no statistically significant differences from either in these two PCs (Fig. 2, SI Fig. 2). Morphologically, specimens located in the positive end of PC1 have wider rami and a more projecting anterior alveolar region. Specimens which are in the positive end of PC2 have more inferiorly positioned coronoid processes, more flexed posterior margins of the ramus and less projecting gonia. Visual assessment of Fig. 2 also suggests more diversity in the Chalcolithic Iberian sample than in others. This assessment is supported by the analysis of shape disparity, which shows that the within-group Procrustes distances are greater in Chalcolithic Iberia than in all other groups (Mesolithic Iberia = 0.0039, Neolithic Iberia = 0.0042, Late Neolithic—Chalcolithic Iberia = 0.0030, Chalcolithic Iberia = 0.0062, Chalcolithic Levant = 0.0034; Table 2).

**Table 2:** Results of the analysis of shape disparity of groups.

	Mesolithic Iberia	Neolithic Iberia	Late Neolithic—Chalcolithic Iberia	Chalcolithic Iberia	Chalcolithic Levant
Mesolithic Iberia		0.0003	0.0010	0.0023	0.0005
Neolithic Iberia	0.6771		0.0012	0.0020	0.0008
Late Neolithic—Chalcolithic Iberia	0.3262	0.2525		0.0032	0.0004
Chalcolithic Iberia	**0.0004**	**0.0094**	**0.0030**		0.0028
Chalcolithic Levant	0.2125	0.2041	0.6844	**0.0001**	

Pairwise differences between variances (upper triangle of the matrix) and the *p*-values for statistical testing of pairwise differences between groups (lower triangle of the matrix; highlighted in bold when significant). Note that the Neolithic Levantine sample was not included because it comprised only one specimen.

**Figure 3:**
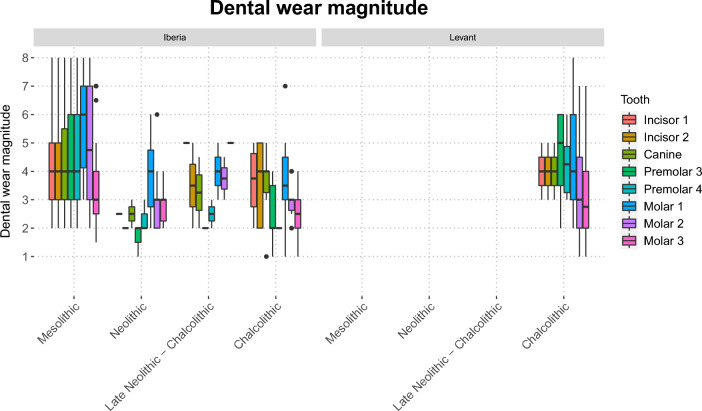
Dental wear magnitude, scored according to B.H. Smith ^47^, across Mesolithic, Neolithic and Chalcolithic groups. Results for the Iberian and Levantine samples are shown separately. In the Levantine sample there are no Mesolithic or Late Neolithic—Chalcolithic specimens, and only one Neolithic specimen.

The total number of teeth in several of the samples was small primarily because of post-mortem loss of anterior teeth (Table SI 1), but comparison of all tooth types indicated that tooth wear was heavier in the Iberian Mesolithic group than in the more recent Iberian samples (Fig. 3, SI Fig. 3). More meaningful comparisons of the magnitude of tooth wear were made on the first and second molars, where samples sizes were larger. The results showed that the magnitude of wear in first molars was significantly greater in the Iberian Mesolithic than in most other samples (SI Fig. 3). In the second molar, differences in dental wear followed the same pattern, but statistically significant differences were only present between the Iberian Mesolithic and Chalcolithic samples from Iberia and the Levant (SI Fig. 3).

**Figure 4:**
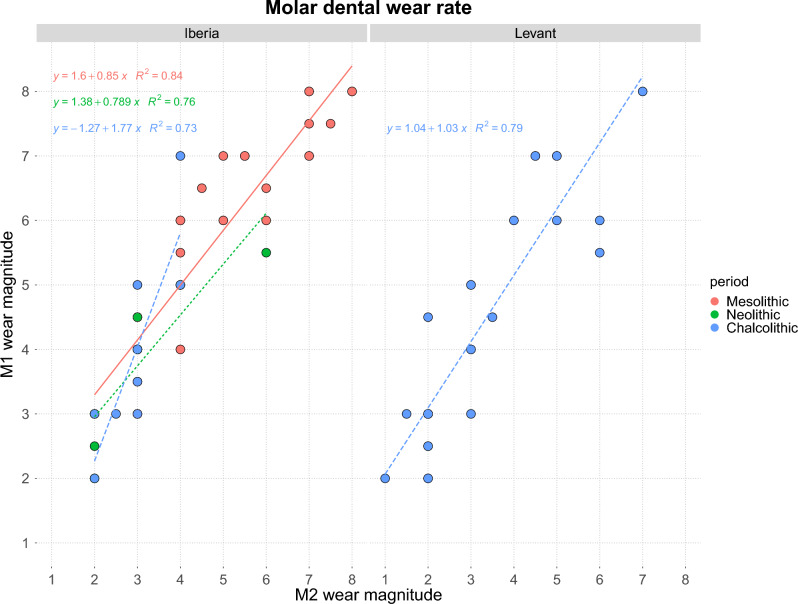
Rate of dental wear in different periods. Results are shown separately for the Iberian and Levantine samples. The Levantine Neolithic sample was not included in the analysis because it comprised only one individual.

The differences in severity of wear of paired first and second molars is shown in Fig. 4. The wear scores plotted for the different groups varied, with few Iberian Neolithic or Chalcolithic first molars scoring 5 or more. This contrasted with the Iberian Mesolithic and Levantine Chalcolithic molars where more severe wear scores were common. This was reflected in the slope of the regression lines that were plotted. These were steepest in the Iberian and Levantine Chalcolithic samples. However, no statistically significant differences were found between groups (SI Fig. 4).

Regressing mandibular shape (as assessed by the scores of the first 29 PCs) against first and second molar dental wear magnitude scores yields very low R^2^ values and non-significant p values in most cases (Table SI 2), thus revealing little impact of masticatory function (as assessed by wear magnitude) on mandibular morphology.

## Discussion

The results obtained show no difference in mandibular size between samples, but significant shape differences between Mesolithic Iberian and almost all other populations, as well as between the Levant Chalcolithic and all other groups when considering the PCs explaining ~ 95% of the variance. The first two PCs (~ 27% variance) show that the Iberian Neolithic to Chalcolithic samples overlap with both the Iberian Mesolithic and Levantine Chalcolithic samples (which are statistically significantly different from each other) and show no significant differences when compared to any of all other populations. This intermediate position is expected given the relationship between mandibular morphology and population history^19,44,45^, and accords with the aDNA findings demonstrating diffusion of Neolithic Near Eastern populations into Iberia and some degree of subsequent admixture with the local pre-existing population^2–4,8,15,16^. Overall, these results confirm our prediction of rejection of the null hypothesis of no differences between Mesolithic Iberia and the Chalcolithic Levant, and that post-Mesolithic Iberians are morphologically intermediate between these groups.

Moreover, our results for severity of dental wear in molars show highly significant differences between the Iberian Mesolithic, more recent Iberian and the Levantine samples. This is most severe in the Iberian Mesolithic followed by the Levantine Chalcolithic and least severe in the Iberian Neolithic and Chalcolithic sample. Because dental wear relates to the material properties of foodstuffs^24,47–54^, this suggests limited differences in the material properties of the diets between these two former populations. Interestingly, visual assessment of morphological differences across specimens along PC1 and 2 suggests that the Iberian Mesolithic specimens are generally more gracile than the Chalcolithic Levant mandibles (Fig. 2) despite the heavier dental wear of the Iberian Mesolithic sample that would be expected to affect biomechanical adaptation of the mandible^55–61^. Moreover, and contrary to our original prediction, regressing PC scores against dental wear revealed very little relationship between the two, thus showing that dental wear magnitude only explains a very minor proportion of shape variance. Altogether this suggests that population history (rather than masticatory mechanics) plays the most important role on the differences seen in mandibular morphology in our study.

Several previous studies support gradual dietary changes throughout the Neolithic and Chalcolithic, i.e., after the introduction of farming (and therefore of different material properties of foodstuffs). Godinho, et al.^25^ found evidence of decreased dental wear in Iberia in the transition from the Neolithic to the Chalcolithic, possibly related to agricultural intensification. Dietary stable isotope analysis in Iberia also supports a gradual change from the early Neolithic to the Chalcolithic period which was related to agricultural intensification^30^. Lastly, Quagliariello, et al.^34^ found evidence of changes in the oral microbiome during the Neolithic—Chalcolithic transition that they attributed to changes in diet. Our results show overlap of the Iberian Neolithic to Chalcolithic samples and so do not detect a gradual change in mandibular form throughout this chronological span. This may be consistent with the greater impact of population history on mandibular morphology. Although our results for dental wear do not detect meaningful changes in dental wear between the Neolithic and the Chalcolithic, the dental sample is very small (in some cases n = 1), precluding well supported considerations.

Interestingly, we detected larger morphological variance (i.e., disparity) in the Iberian Chalcolithic than in other samples. This finding is of particular relevance because of the presence of dental and skeletal traits in this period in Iberia that are more common in African populations^62,63^. Moreover, aDNA studies show some contact between Chalcolithic Iberians and North Africans^15^, and the presence of raw materials originating in regions as distant as Asia and North Africa relate to the expansion of Chalcolithic trading networks^64–69^. Higher levels of mobility during this period are also reflected in the presence of non-local individuals in multiple regions^70–72^. We thus hypothesize that the large morphological disparity detected in the Iberian Chalcolithic mandibles may relate to increased contact with exogenous populations.

The population and dietary changes in the Mesolithic–Neolithic transition are also associated with other socio-economic-biological changes^17^. Specifically, the Mesolithic forager economy is typically associated with high levels of mobility that are reflected in high bending strength of the femur and tibia^73–76^. Interestingly, Iberian Mesolithic populations are reported to have limited bending strength of the lower limb when compared to later populations from the Bronze Age^77^. While this contrasts with the overall pattern of lower limb morphology in this timeframe, these are late Mesolithic populations that are already somewhat sedentary and occupied an area with low topographic relief, which is reflected in the cross-sectional anatomy of the bones of the lower limb^24,77^.

Despite our interpretation that (i) mandibular shape differences between Iberia and the southern Levant are likely mostly due to contrasting population histories, and (ii) the overlap shown by the two most relevant PCs between the Iberian Neolithic to Chalcolithic group to all others is probably related to further contacts over time in the wake of increased trade and population movements, the following caveats and limitations should be considered.

The single Neolithic Levantine specimen from Abu Gosh that was used in this study dates to the final stage of the Neolithic period, so it is not surprising that it falls within the range of variation of the Levantine Chalcolithic sample. In this it conforms to results of previous studies that showed no differences between southern Levantine Neolithic and Chalcolithic mandibular morphology^21^. Moreover, aDNA studies^78^ have shown that Neolithic and Chalcolithic southern Levantine populations are directly related. Thus, although earlier Neolithic southern Levantine populations would be better suited to examine the relationship between Neolithic-Chalcolithic Iberian mandibular morphology and population admixture between Mesolithic Iberians and migrating Near Eastern populations, the southern Levantine Chalcolithic sample appears a generally reliable proxy.

This study compares Iberian and southern Levantine samples. aDNA studies have shown that Iberian Neolithic-Chalcolithic populations are closer to Anatolian and European Neolithic populations than to those from the southern Levant^7,15,79^. This reflects the “dilution” of the initial Near Eastern phenotype through admixture with other Mediterranean populations over time and space as populations spread westward. Thus, although a relationship between the recent Prehistoric southern Levantine and Iberian samples is to be expected, it is impacted by the complexity of the westward expansion of the Neolithic to Europe. Thus, future studies should expand the non-Iberian sample and include more southern Levantine specimens from the Neolithic and Chalcolithic to ensure there are no meaningful differences between these chronologically distinct groups, and that the shape differences detected in this study between the Iberian and southern Levantine samples do not result from sampling bias.

Despite these caveats for future research, our study documents significant mandibular shape differences between the Iberian Mesolithic and the southern Levant Chalcolithic, with the Iberian Neolithic to Chalcolithic samples occupying an intermediate position between them. Further, we found little relationship between mandibular shape and dental wear, limited differences in dental wear (and so function) between the Mesolithic Iberians and the Chalcolithic southern Levantine groups, and larger differences relative to post-Mesolithic Iberians. Altogether, this is consistent with demic diffusion of Near Eastern Neolithic populations. Thus, admixture with incoming Neolithic populations may have contributed to the extent and pattern of changes, as well as increased variation, seen in the teeth and jaws of Iberian Neolithic and Chalcolithic populations. Moreover, dietary differences appear to have had limited impact on the observed morphological differences across our samples.

## Materials and methods

### Sample

**Figure 5:**
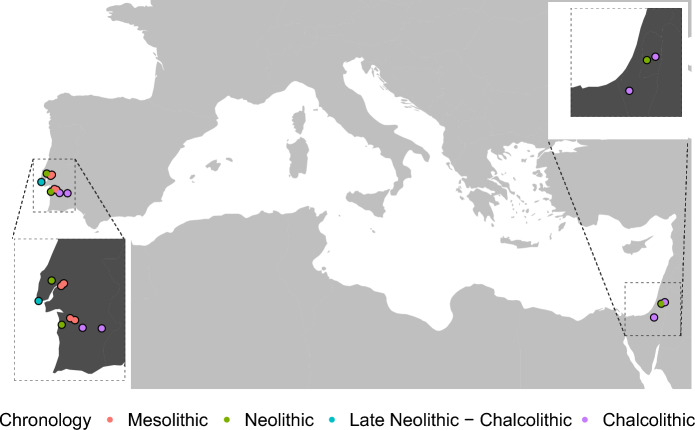
Map with geographical and chronological origin of the samples (not-to-scale). The map was created using the *rnaturalearth*^91^ and *ggplot2*^92^ packages of the R software.

In this study we examined 101 human mandibles originating from southwestern Iberia (central and southern Portugal) and the southern Levant (Judean desert), that chrono-culturally span the Mesolithic to Chalcolithic periods (Fig. 5 and Table 3). Only adult specimens were selected (i.e., specimens at least 18 years old). The reason is that mandibular form changes dramatically throughout growth and development^80,81^, and so it is critical to ensure that age-related morphological differences do not overshadow group-related differences, which were the focus of this study. Mandibular morphology is also impacted by sexual dimorphism^82–86^. Yet, morphological based sex estimation is most reliable when using the hip bones and the full skull^87,88^ (which is not possible in most cases with these samples; see details below). Previous studies using identified collections report correct classification rates of as low as 68%^85,86^ when estimating sex based on mandibles alone, showing potential limitations of this approach. Moreover, morphological sex differences are not explained exclusively by size (both isometric and allometric) differences^83,89,90^, limiting the efficacy of removing size related shape differences to exclude the effects of sexual dimorphism from ensuing analyses. Thus, although morphological based sex estimation was undertaken using only the mandibles, this data was not included because it is not conclusive and so may bias the results of the study.

**Table 3:** Number of individuals sampled per chronological and geographic region.

Chronology	Iberia	Levant	Total
Mesolithic	37	0	37
Neolithic	11	1	12
Late Neolithic—Chalcolithic	4	0	4
Chalcolithic	12	36	48
Total	64	37	101

### Age at death assessment

Age at death was estimated based on dental development and eruption using the standards of AlQahtani, Hector and Liversidge^93^. To that end, macroscopic observation of these parameters was supplemented by Computed Tomography (CT) scan-based assessment of tooth growth and development whenever possible (see below), allowing observation of non-erupted teeth and roots. No other age-at-death proxies were used because the nature of the samples does not allow conclusive identification and analysis of skeletal elements from the same individual other than the mandible. This is because several Mesolithic samples were affected by post-excavation mixing^94^ and the post-Mesolithic Iberian and Levantine samples originated from collective, commingled funerary contexts^95–99^. Alternatively, tooth wear-based estimation could have been used due to the relationship between wear magnitude and age^54,100,101^. However, since it is also impacted by diet, and in some cases paramastication, it is not a reliable age-at-death proxy across different populations^102–104^.

### Digitization and morphological analysis

Most Iberian specimens were CT scanned using a Toshiba Astelion scanner with an algorithm optimized for scanning bone (120 kV, voxel size 0.348 × 0.348 × 0.3, revolution time 0.75 s, spiral pitch factor 0.94) at the Faculty of Veterinary Medicine of the University of Lisbon. A smaller number of the Iberian specimens was digitized using an Einscan Pro 2X Plus surface scanner. Levantine specimens were digitized using the same scanning algorithm used for scanning the Iberian specimens (120 kV., voxel size 0.307 × 0.307 × 0.6 mm., revolution time 0.75 s, spiral pitch factor 0.94) at the Koret School of Veterinary Medicine (Rehovot, Israel).

In the case of the CT-scanned mandibles, segmentation was performed in 3D Slicer^105^ using standard protocols^106–109^. Specimens that were fragmented were pieced together virtually^110^. Extraction of landmark (LM) coordinates ensued using a set of 21 anatomical LMs (Table 4). In incomplete specimens, the function *estimate.missing* of the R package *geomorph*^111^ was used to estimate the original location of the missing LMs geometrically (i.e., Thin Plate Splines rather than multivariate regression based reconstruction was used). To that end, population specific reference mean specimens were used to reconstruct incomplete specimens (i.e., the mean of the complete Iberian Mesolithic mandibles was used as reference to estimate the missing regions of the incomplete Iberian Mesolithic mandibles; similarly, Neolithic Iberian specimens were used as reference to reconstruct incomplete specimens from that group), although a previous sensitivity study showed negligible to no differences in the outcome of population specific and non-population specific reconstruction of modern human mandibles^46^. The former reconstruction approach was chosen because previous studies also show that using inadequate references (i.e., references from populations that are morphologically meaningfully different from the target reconstructed specimens) may lead to large estimation errors^112–114^. Even though 66 hemi-mandibles were partially reconstructed using this approach (Table SI 3), many other specimens were excluded because reconstruction of excessively incomplete mandibles (i.e., with more than ~ 5 missing LMs when using the LM set of this study) may result in meaningful reconstruction error and so in biased results^115^.

**Table 4:** Landmarks used in this study to extract configurations of 3D coordinates.

# LM	LM name	Landmark definition
1	Gnathion	Midline of the inferior border of the mandible
2	Infradentale	On the anterior alveolar ridge, between anterior incisors
3	Linguale	Genial tubercle: in case of a single tubercle, on its tip; in case of two, midpoint between them
4	Orale, mandible	On the posterior alveolar ridge between the anterior incisors
5	Pogonion	Most anterior point of mandibular symphysis
6	C-P3	On the anterior alveolar ridge between canine and first premolar
7	P4-M1	On the anterior alveolar ridge between second premolar and first molar
8	M1-M2	On the anterior alveolar ridge between first and second molar teeth
9	Mental Foramen Anterior	Anterior point of mental foramen
10	Ramus Root	On the anterior rim of the ramus (placed on the level of the alveolar ridge)
11	Gonion	A point on the projection of the bisection of the mandibular angle
12	Condyle, lateral	From a superior view, the lateral point on the condyle
13	Condyle, midpoint	From a superior view, a point in the center of the condyle
14	Condyle, medial	From a superior view, the medial point on the condyle
15	Sigmoid Notch	Mandible is positioned in the mandibular plane in a lateral view, then the lowest point of the mandibular notch is marked
16	Coronoid process	Tip of the coronoid process
17	Mandibular Foramen, inferior	Most inferior point of the mandibular foramen
18	Alveolous, lingual posterior	From a superior view, the most posterior point on the lingual alveolar process
19	Condyle, anterior	A point on the antero-superior aspect of the mandibular notch (on the condyle)
20	Condyle, posterior	The center of the condyle from a posterior view
21	Ramus, posterior	Posteriormost point of the ramus that is in line with the ramus root

Standard GM analyses using the full sample followed reconstruction of the incomplete specimens in the R *geomorph* package^111^ and EVAN toolbox^116,117^. First, Generalized Procrustes Analysis (GPA) was used to superimpose all landmark configurations and remove the effects of location, size and orientation on the raw coordinates^118,119^. The resulting shape variables were then used to examine morphological variance and hypothetical similarities and/or differences between groups. To that end, Principal Component Analysis (PCA) was used to reduce dimensionality and examine shape differences between specimens, which were visualized warping a surface along the relevant PCs. Size differences between specimens and groups were assessed using centroid size.

Specimens were grouped based on chronology and region (i.e., Mesolithic Iberia, Levantine Chalcolithic, etc.). Potential differences in size (i.e., centroid size) were examined using the Kruskal–Wallis test. Shape differences were first tested using a nonparametric test Permutational Multivariate ANOVA (PERMANOVA) to assess potential multivariate shape differences in the different groups^120^. This was based on the first 29 PCs, which explain ~ 95% of the total variance and was implemented in Past^121^ using 10,000 permutations. The Kruskal–Wallis test (followed by post-hoc tests) was used to test for differences in the two first PCs, which were used together with surface warping to visualize shape differences across the groups. Kruskal–Wallis tests were implemented using the R package *ggstatsplot*^122^. The use of non-parametric testing was necessary for centroid size and PCs 1 and 2 after the Shapiro–Wilk's test revealed that the ANOVA assumption of normality of residuals was not met for size, and Levene's test of homogeneity of variances was not met for shape. These tests, along with the Durbin Watson test for independence of residuals, were carried out in the *car* R package^123^. Lastly, the *morphol.disparity* function of the *geomorph* package^111^ was used to examine hypothetical differences in shape variance across groups (i.e., disparity using Procrustes distances).

### Relationship between dental wear magnitude and mandibular morphology

Because dental wear relates to diet and masticatory function, we used it as a proxy to examine differences in the material properties of foodstuffs eaten by the different groups examined. To that end, dental wear magnitude was scored for each tooth according to the eight stage ordinal scale of B.H. Smith ^47^. Previous studies (based on part of the sample used in this present study), have shown that ante and postmortem tooth loss had resulted in the absence of many teeth^25^. This is especially pervasive in the anterior dentition, in which only 28/101 (27.72%) of lower central right incisors were present, compared to 75/101 (74.26%) of lower left first molars (Table SI 1). To mitigate this effect, antimeres were pooled, and wear scores averaged. The wear score of single teeth was used whenever antimeres were absent. As in other studies using this approach^25,103,124^, this procedure ensued after we found no statistically significant differences in the magnitude of wear between antimeres, using Wilcoxon pairwise comparisons in R.

Even though dental wear was used in this study as a proxy for differences in diet and masticatory function, it is also influenced by age^54,100,101^. Regrettably, the nature of the samples did not allow age-at-death estimation based on skeletal elements other than the mandible. Thus, in addition to quantifying dental wear of individual teeth, we also compared rates of dental wear of paired first and second molars. This approach was used because it has been demonstrated to be age independent and used in other studies to compare the rate at which teeth are worn, which should relate to the material properties of foodstuffs processed intra-orally^54,99,125^.

Dental wear was scored by the same observer (RMG), preventing inter-observer error. Although not all teeth were observed multiple times, a previous study with a sub-sample (n = 412) of the one used in this study showed no scoring differences between repeated observations by the same observer^126^. Dental wear scores of paired first and second molars were plotted using the *ggplot2* package of R^92^. Statistical testing of dental wear magnitude differences between groups were carried out using the non-parametric Kruskal–Wallis test, together with post-hoc tests, which were implemented using the *ggstatsplot* R package^122^. Non-parametric testing was necessary because ANOVA assumptions (i.e., homogeneity of variance, normal distribution and independence of residuals) were not met.

To explore the relationship between dental wear and mandibular morphology, multiple regression was used to regress the scores of the first 29 PCs against wear magnitude scores of the first and second molars (which yielded the largest samples and statistically significant differences between groups) in R. The regression analysis was undertaken independently for each group.

### Supplementary Information

Supplementary Information.

## Data Availability

The data that support this study is available from the corresponding author upon reasonable request.
