# Supplementary material for: Mandibular morphology and the Mesolithic–Neolithic transition in Westernmost Iberia
Source: Sci Rep. 2023 Oct 3;13:16648. doi: 10.1038/s41598-023-42846-z (PMC10547775; doi:10.1038/s41598-023-42846-z)
Supplement: Supplementary file 1 — Supplementary Information. [file 41598_2023_42846_MOESM1_ESM.pdf]

Mandibular morphology and the Mesolithic–Neolithic transition in Westernmost Iberia – Supplementary information

Ricardo Miguel Godinho, Cláudia Umbelino, António Carlos Valera, António Faustino de Carvalho, Nuno Bicho, João Cascalheira, Célia Gonçalves, Patricia Smith

Table SI 1: Inventory of teeth present and used in the dental wear magnitude analysis of this study.

**Site**	**Period**	**fdi.31**	**fdi.32**	**fdi.33**	**fdi.34**	**fdi.35**	**fdi.36**	**fdi.37**	**fdi.38**	**fdi.41**	**fdi.42**	**fdi.43**	**fdi.44**	**fdi.45**	**fdi.46**	**fdi.47**	**fdi.48**	**Total**
Abu Gosh	Neolithic	1	1	1	1	1	0	1	1	1	1	1	1	1	1	1	1	15
Algar do Bom Santo	Neolithic	1	1	1	1	2	4	3	2	1	0	1	1	0	2	3	3	26
Arapouco	Mesolithic	1	1	1	1	1	1	2	2	2	1	0	1	2	2	1	1	20
Cabeço da Amoreira	Mesolithic	1	1	1	1	1	1	1	1	1	1	1	1	1	1	1	1	16
Cabeço da Arruda	Mesolithic	4	5	8	8	9	9	8	7	5	7	9	9	8	8	7	5	116
Cabeço de Pez	Mesolithic	1	1	1	1	0	1	0	0	1	0	0	1	1	1	0	0	9
Cova da Onça	Mesolithic	1	1	1	1	1	1	1	1	1	1	1	0	1	1	1	1	15
Gruta do Zambujal	Neolithic	0	0	0	1	0	5	1	0	0	1	1	0	0	6	3	1	19
Grutas de Melides	Neolithic	0	0	0	1	1	1	1	0	0	0	0	0	1	1	1	1	8
Grutas do Poço Velho	Late Neolithic - Chalcolithic	1	0	1	0	0	1	1	1	0	2	2	1	2	2	2	0	16
Moita do Sebastião	Mesolithic	12	14	15	17	15	16	16	11	11	15	14	17	16	16	15	11	231
Monte da Guarita 2	Chalcolithic	2	3	5	3	0	5	6	5	1	1	3	3	0	2	4	3	46
Monte do Carrascal 2	Chalcolithic	2	1	1	2	2	5	4	2	1	1	1	1	1	3	3	1	31
Shiqmim	Chalcolithic	2	2	2	2	1	2	2	1	2	2	2	2	1	1	1	1	26
Vale de Romeiras	Mesolithic	0	1	2	2	2	1	1	2	1	1	2	1	1	2	2	2	23
Wadi Makkukh	Chalcolithic	1	0	0	0	1	22	23	14	0	0	0	2	4	21	24	12	124
Total		30	32	40	42	37	75	71	50	28	34	38	41	40	70	69	44	741

Table SI 2: Results of regressing the scores of the first 29 PCs against dental wear magnitude of the first and second molars.

	Mesolithic Iberia				Neolithic Iberia				Chalcolithic Iberia				Chalcolithic Levant			
	R2	Adjusted R2	F statistic	p	R2	Adjusted R2	F statistic	p	R2	Adjusted R2	F statistic	p	R2	Adjusted R2	F statistic	p
PC1	0.1268	0.0596	1.8870	0.1716	0.1295	-0.1607	0.4462	0.6597	0.1295	-0.1607	0.4462	0.6597	0.0862	0.0101	1.1320	0.3390
PC2	0.0859	0.0156	1.2220	0.3109	**0.6795**	**0.5726**	**6.3590**	**0.0329**	**0.6795**	**0.5726**	**6.3590**	**0.0329**	0.1108	0.0367	1.4950	0.2444
PC3	0.1222	0.0547	1.8100	0.1836	0.5445	0.3927	3.5870	0.0945	0.5445	0.3927	3.5870	0.0945	0.1076	0.0332	1.4460	0.2553
PC4	0.1727	0.1091	2.7140	0.0850	0.1326	-0.1566	0.4585	0.6527	0.1326	-0.1566	0.4585	0.6527	0.1696	0.1004	2.4500	0.1076
PC5	**0.3879**	**0.3408**	**8.2390**	**0.0017**	0.2229	-0.0362	0.8603	0.4693	0.2229	-0.0362	0.8603	0.4693	0.2186	0.1535	3.3570	0.0518
PC6	0.0100	-0.0661	0.1317	0.8772	0.1227	-0.1698	0.4194	0.6753	0.1227	-0.1698	0.4194	0.6753	0.0517	-0.0273	0.6542	0.5289
PC7	0.1019	0.0328	1.4740	0.2475	0.3093	0.0791	1.3430	0.3295	0.3093	0.0791	1.3430	0.3295	0.0469	-0.0325	0.5906	0.5618
PC8	0.0591	-0.0133	0.8164	0.4530	0.0718	-0.2375	0.2322	0.7996	0.0718	-0.2375	0.2322	0.7996	0.1710	0.1019	2.4750	0.1054
PC9	0.1341	0.0675	2.0130	0.1539	0.0115	-0.3180	0.0350	0.9658	0.0115	-0.3180	0.0350	0.9658	0.0680	-0.0096	0.8759	0.4294
PC10	0.0090	-0.0672	0.1185	0.8888	0.3479	0.1305	1.6000	0.2774	0.3479	0.1305	1.6000	0.2774	0.0711	-0.0064	0.9180	0.4129
PC11	0.0952	0.0256	1.3670	0.2725	0.4285	0.2380	2.2490	0.1867	0.4285	0.2380	2.2490	0.1867	0.0066	-0.0761	0.0803	0.9231
PC12	0.0074	-0.0689	0.0970	0.9078	0.0395	-0.2806	0.1234	0.8861	0.0395	-0.2806	0.1234	0.8861	0.0111	-0.0714	0.1341	0.8751
PC13	0.0276	-0.0472	0.3688	0.6951	0.0591	-0.2545	0.1885	0.8330	0.0591	-0.2545	0.1885	0.8330	0.0157	-0.0663	0.1912	0.8272
PC14	0.1932	0.1312	3.1130	0.0614	0.5062	0.3415	3.0750	0.1204	0.5062	0.3415	3.0750	0.1204	0.0613	-0.0169	0.7835	0.4682
PC15	0.0512	-0.0218	0.7009	0.5053	0.0951	-0.2065	0.3154	0.7409	0.0951	-0.2065	0.3154	0.7409	0.0007	-0.0825	0.0089	0.9911
PC16	0.0326	-0.0418	0.4383	0.6498	0.2415	0.2415	0.9551	0.4364	0.2415	-0.0114	0.9551	0.4364	0.1406	0.0690	1.9630	0.1623
PC17	0.0509	-0.0220	0.6977	0.5068	0.1667	-0.1111	0.5999	0.5787	0.1667	-0.1111	0.5999	0.5787	0.0310	-0.0498	0.3839	0.6853
PC18	0.1939	0.1319	3.1270	0.0607	0.0123	-0.3169	0.0374	0.9635	0.0123	-0.3169	0.0374	0.9635	**0.2276**	**0.1633**	**3.5370**	**0.0451**
PC19	0.0373	-0.0368	0.5035	0.6102	0.1515	-0.1313	0.5358	0.6108	0.1515	-0.1313	0.5358	0.6108	0.0559	-0.0228	0.7102	0.5016
PC20	0.1632	0.0989	2.5360	0.0986	0.2647	0.0196	1.0800	0.3976	0.2647	0.0196	1.0800	0.3976	0.0048	-0.0781	0.0581	0.9437
PC21	0.0875	0.0173	1.2470	0.3040	0.1737	-0.1018	0.6304	0.5643	0.1737	-0.1018	0.6304	0.5643	0.1537	0.0831	2.1790	0.1351
PC22	0.0473	-0.0260	0.6454	0.5327	0.1379	-0.1495	0.4797	0.6408	0.1379	-0.1495	0.4797	0.6408	0.0343	-0.0462	0.4256	0.6582
PC23	0.0312	-0.0434	0.4184	0.6625	0.0378	-0.2829	0.1179	0.8908	0.0378	-0.2829	0.1179	0.8908	0.0085	-0.0742	0.1024	0.9031
PC24	0.0157	-0.0600	0.2073	0.8141	0.3758	0.1677	1.8060	0.2432	0.3758	0.1677	1.8060	0.2432	0.0020	-0.0812	0.0238	0.9765
PC25	**0.2319**	**0.1728**	**3.9250**	**0.0324**	**0.6724**	**0.5632**	**6.1580**	**0.0352**	**0.6724**	**0.5632**	**6.1580**	**0.0352**	0.0936	0.0181	1.2400	0.3074
PC26	0.0350	-0.0392	0.4721	0.6289	0.1678	-0.1095	0.6051	0.5763	0.1678	-0.1095	0.6051	0.5763	0.0854	0.0092	1.1210	0.3425
PC27	0.0582	-0.0143	0.8031	0.4587	0.2888	0.0517	1.2180	0.3597	0.2888	0.0517	1.2180	0.3597	0.0545	-0.0243	0.6913	0.5106

PC28	0.0870	0.0167	1.2380	0.3065	0.1483	-0.1356	0.5224	0.6178	0.1483	-0.1356	0.5224	0.6178	0.1192	0.0458	1.6240	0.2180
PC29	0.0210	-0.0543	0.2788	0.7589	0.2994	0.0659	1.2820	0.3439	0.2994	0.0659	1.2820	0.3439	0.1120	0.0380	1.5130	0.2405
Min	0.0074	-0.0689	0.0970	0.0017	0.0115	-0.3180	0.0350	0.0329	0.0115	-0.3180	0.0350	0.0329	0.0007	-0.0825	0.0089	0.0451
Max	0.3879	0.3408	8.2390	0.9078	0.6795	0.5726	6.3590	0.9658	0.6795	0.5726	6.3590	0.9658	0.2276	0.1633	3.5370	0.9911
Mean	0.0923	0.0225	1.4693	0.4221	0.2358	-0.0102	1.2869	0.5166	0.2358	-0.0189	1.2869	0.5166	0.0800	0.0033	1.1091	0.4742

Table SI 3: Summary of the number of missing landmarks per specimen.

**Missing LMs**	**n**
0	35
1	20
2	22
3	14
4	9
5	1
Total	101

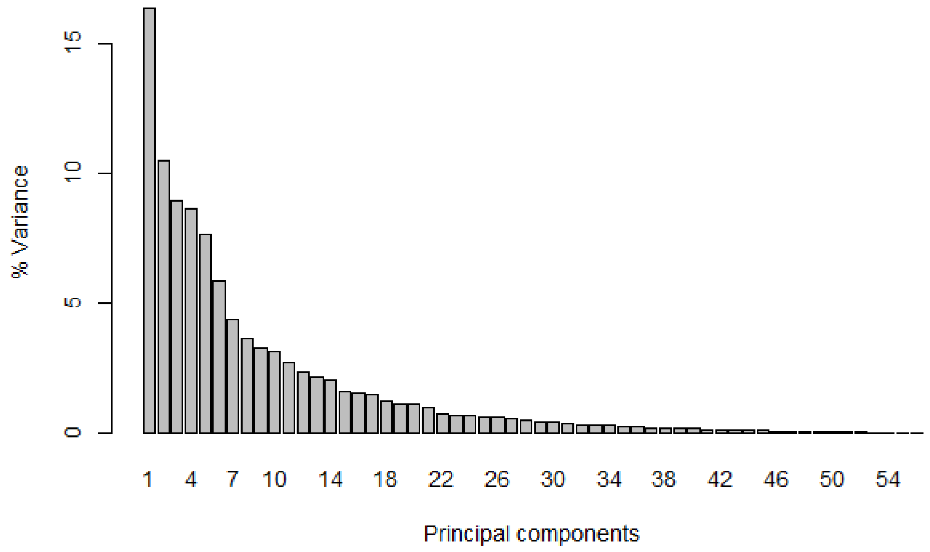

SI Figure 1: Histogram with percentage of variance explained by the principal components of the shape analysis.

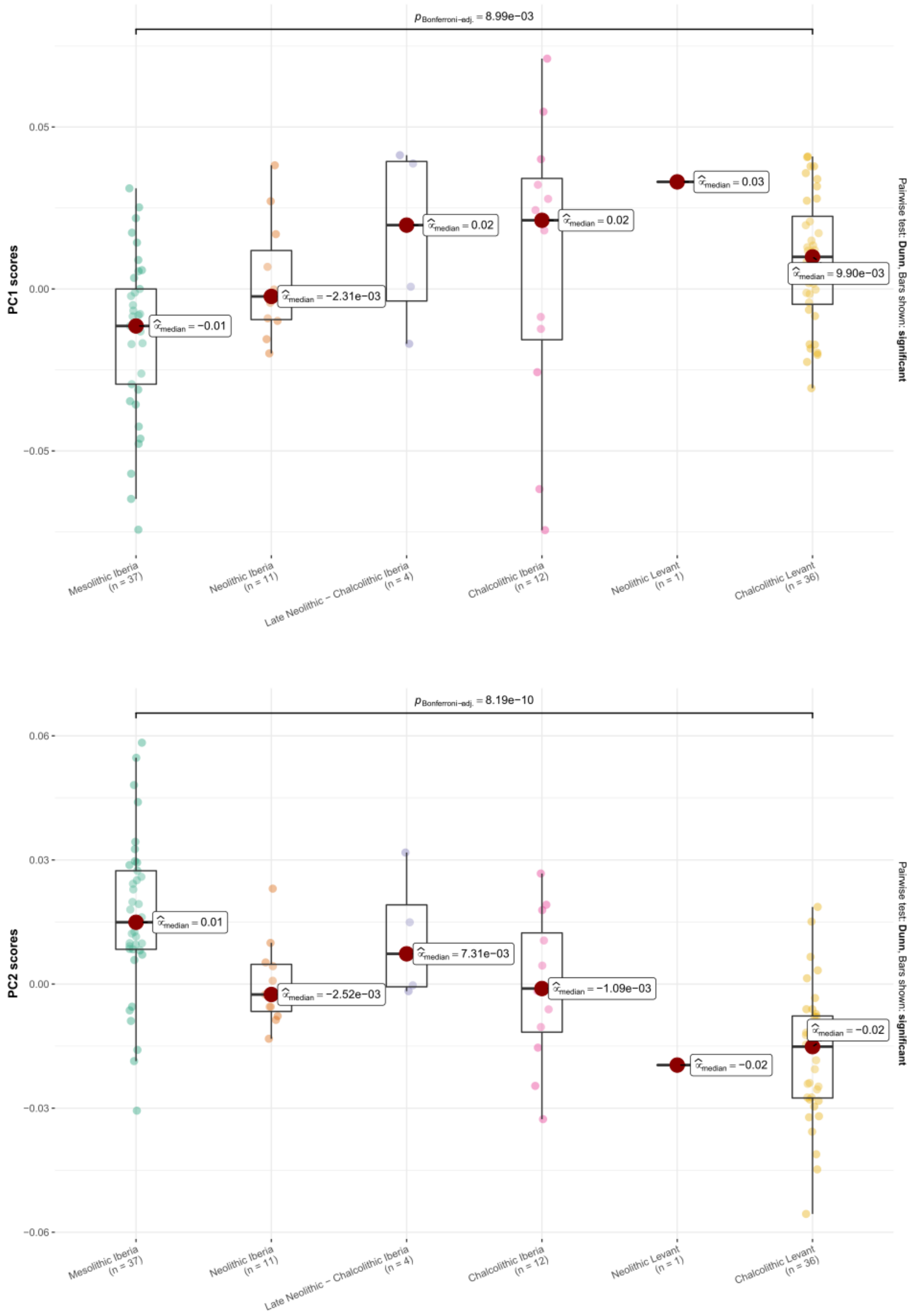

SI Figure 2: Statistical testing of differences in PC 1 and 2 scores between different groups.

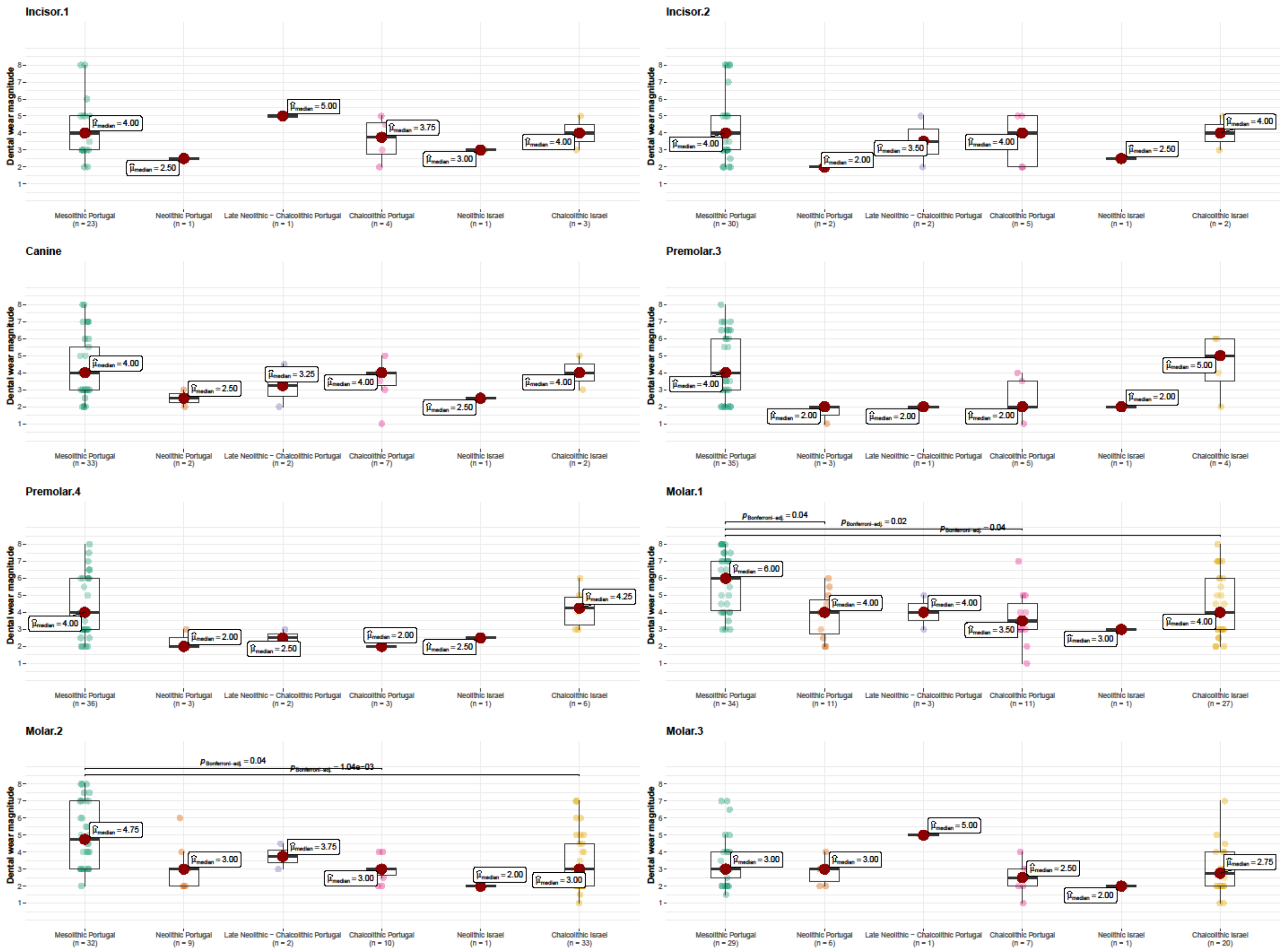

SI Figure 3: Statistical testing of differences in dental wear magnitude. Results are grouped by tooth type and compared across groups.

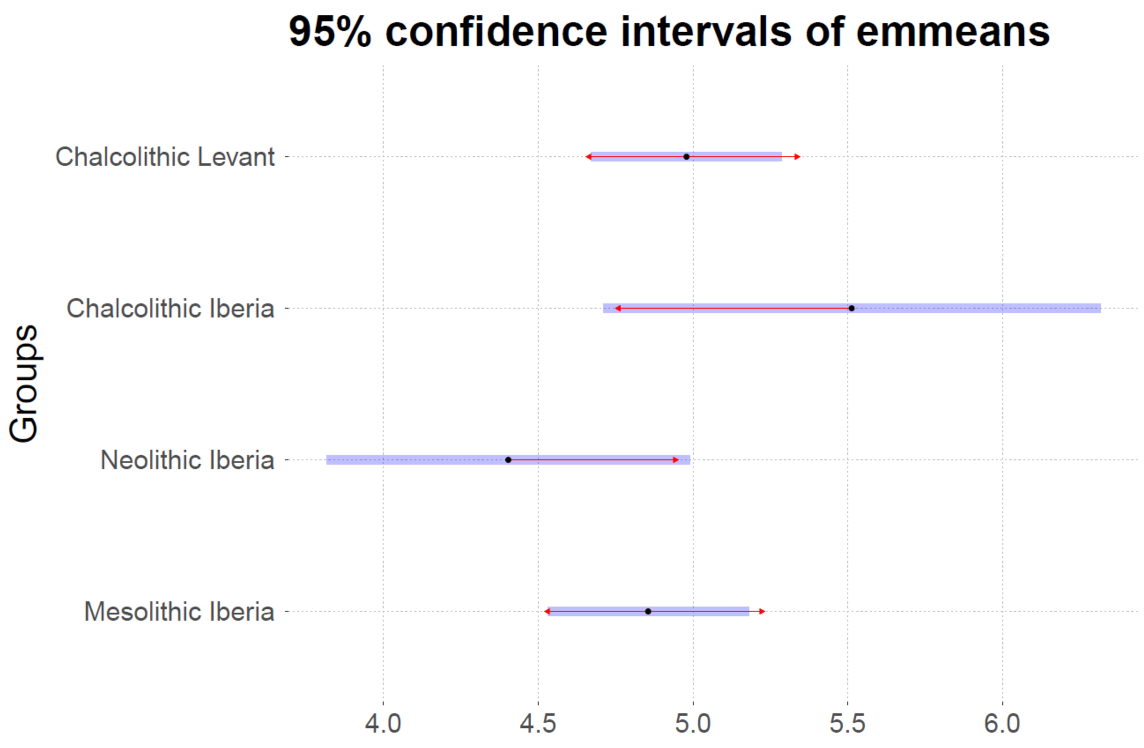

SI Figure 4: Statistical testing for hypothetical differences in rates of wear across the samples. The overlap between all groups reveals no significant differences.
